# α-Mangostin and lawsone methyl ether in tooth gel synergistically increase its antimicrobial and antibiofilm formation effects in vitro

**DOI:** 10.1186/s12903-023-03511-z

**Published:** 2023-11-08

**Authors:** Wipawee Nittayananta, Panjaporn Wongwitthayakool, Teerapol Srichana, Chadaporn Setthanurakkul, Panthakarn Yampuen, Paphawarin Terachinda, Titima Deebunjerd, Jiratchaya Tachapiriyakun

**Affiliations:** 1 Faculty of Dentistry, Thammasat University, Pathum Thani, Thailand; 2 Drug Delivery System Excellence Center, Faculty of Pharmaceutical Sciences, Prince of Songkla University, Hat Yai, Songkhla, Thailand; 3 Department of Pharmaceutical Technology, Faculty of Pharmaceutical Sciences, Prince of Songkla University, Hat Yai, Songkhla, Thailand

**Keywords:** Antimicrobial activity, Antibiofilm, Candidiasis, Dental caries, Lawsone methyl ether, Mangosteen, Periodontitis

## Abstract

**Objectives:**

α-Mangostin (α-MG) and lawsone methyl ether (LME) show antimicrobial and anti-biofilm activities. The objectives of this study were to develop a herbal tooth gel containing α-MG and LME plus fluoride and determine its antimicrobial, anti-biofilm formation, anti-cancer, anti-inflammatory, wound healing, and enamel microhardness effects.

**Methods:**

Antimicrobial assays against *Streptococcus mutans, Porphyromonas gingivalis, and Candida albicans* were performed. The microbes’ ultrastructural morphology was assessed using Transmission Electron Microscopy. The effect on microbial biofilm formation was tested by a broth microdilution. Cell viability was assessed with MTT assay. The anti-inflammatory effect was investigated by measuring inhibition of nitric oxide production. Enamel microhardness was measured via Vickers microhardness testing. The enamel chemical composition was investigated with Fourier Transform Spectrometer. The enamel surface morphology and fluoride content were examined by Scanning Electron Microscopy and Energy Dispersive X-ray Spectroscopy.

**Results:**

The results show synergistic effects of α-MG and LME on antimicrobial activity and antibiofilm formation without cytotoxicity at a therapeutic dose. At a higher dose, the tooth gel inhibited proliferation of cancer cell line. Enamel microhardness was increased after brushing with the tooth gel plus fluoride. A large amount of fluoride was detected on the enamel surface.

**Conclusion:**

The tooth gel containing α-MG and LME synergized its antimicrobial activity and antibiofilm formation and inhibited oral cancer cell proliferation. Incorporating fluoride into the tooth gel increased enamel microhardness. Thus, the herbal tooth gel containing α-MG and LME plus fluoride may be useful for preventing dental caries and promoting oral health.

**Supplementary Information:**

The online version contains supplementary material available at 10.1186/s12903-023-03511-z.

## Introduction

Infectious diseases, including dental caries, periodontitis, and candidiasis are common oral health problems caused by biofilms, a structured community of microbes embedded in a self-organized matrix of extracellular polysaccharides [1]. Caries results from enamel/dentin demineralization due to acid produced by cariogenic bacteria, such as *Streptococcus mutans (S. mutans*), while periodontitis is caused by anaerobic bacteria, e.g., *Porphyromonas gingivalis (P. gingivalis)* [2, 3]. Oral candidiasis is frequently observed in immunocompromised patients and denture wearers, and is caused by *Candida albicans,* i.e., *C. albicans* [4].

Tooth brushing with fluoride toothpaste and flossing along with chemical plaque control by mouthwash, such as chlorhexidine, are the methods used to keep the teeth clean and hygienic to control dental caries and periodontal [5]. Different topical and systemic antifungal agents are available for treating oral candidiasis. However, the bitter taste and staining caused by chlorhexidine are limitations in using this mouthwash. In addition, recurrence and drug resistance to *Candida* usually occurs in immunocompromised hosts without complete resolution of the infection [6, 7]. Thus, alternative strategies are needed in managing these common oral diseases.

The extract from the Mangosteen *(Garcinia mangostana* L), a well-known fruit in Southeast Asian countries [8], has bactericidal activity against cariogenic bacteria [9]. It has been used in traditional medicine to treat infections caused by bacteria, viruses, and fungi [10, 11]. α-Mangostin (α-MG), a major xanthone derivative compound isolated from the Mangosteen pericarp extract, disrupts the development of *S. mutans* biofilm [12]. Furthermore, Tangsuksan et al. [13] reported that a soluble mucoadhesive film containing α-MG is effective against *S.* *mutans*, *P.* *gingivalis*, and *C.* *albicans* without significant cytotoxicity in vitro. The film also demonstrated anti-inflammatory activity by inhibiting nitric oxide production in a dose-dependent manner. α-MG has been added in an adhesive paste to prevent dental caries [14] and put in a topical gel to treat chronic periodontitis [15, 16]. Lawsone methyl ether (2-methoxy-1,4-naphthoquinone) (LME) isolated from flowers of *Impatiens balsamina* L. [17] demonstrated potent antifungal and antibacterial activities in LME containing mouthwash [18] and oral spray [10] without significant cytotoxicity. However, no study has incorporated α-MG and LME in tooth gel and determined its bioactivity against these three common oral microorganisms.

Because α-MG and LME possess various bioactivities, we hypothesized that a fluoride tooth gel containing α-MG and LME would demonstrate antimicrobial activity against *S. mutans*, *P. gingivalis, and C. albicans*, and reduce biofilm formation and inflammation. In addition, fluoride in the tooth gel would be released and increase the tooth surface hardness. Thus, this product could potentially prevent dental caries, periodontal disease, and oral candidiasis development. Therefore, in this study, three formulations of tooth gel: 1) α-MG (M), 2) LME (L), and 3) α-MG and LME (M + L), were developed and evaluated for their cytotoxicity, antimicrobial, anti-biofilm formation, and anti-inflammatory activities in vitro. The effect of fluoride in the tooth gel on enamel microhardness was also assessed ex vivo.

## Materials and methods

### Preparation of tooth gels

The tooth gel base was prepared by mixing poloxamer 407 (1 g) in 10 ml water. Sodium fluoride and xylitol were dissolved in 10 ml water. Sodiummetabisulfite and sodium benzoate were dissolved in 10 ml water. The three solutions were mixed together, followed by adding 5 ml propylene glycol to obtain a solution mixture. The 0.2 g menthol was dissolved in 1 ml peppermint water, then slowly added into the solution mixture. Pemulen TR-2 was dispersed in DI water and mixed with the solution mixture. Finally, the colloidal silica was mixed in the solution mixture to obtain the tooth gel and the final volume was adjusted to 100 g with DI water. Mangosteen peel extract (M) (1 g), 1 g LME (L) and 0.5 g M + 0.5 g L was levigated with 500 µl DMSO to obtain a homogeneous tooth gel. Each component (1 g) was mixed with tooth gel to obtain a 1% M tooth gel, 1% L tooth gel, and 1% M + L tooth gel.

The tooth gels were monitored for their physical properties and stability at room temperature and simulated aging using 6 freeze thaw cycles. The physical appearance, color, pH, and homogeneity of the tooth gels were recorded.

### Scanning electron microscopy (SEM)

The surface morphology of the three tooth gel formulations was examined using a scanning electron microscope (SEM Quanta 400; FEI, Brno, Czech Republic). Before scanning, the tooth gel sample was spread on an aluminum stub and was allowed to dry for 3 h until.

### Fourier transform-infrared spectroscopy (FT-IR)

The functional groups present in the tooth gel sample were identified using FT-IR (Perkin-Elmer, MA, USA). A disk was created by compressing the dry potassium bromide. The tooth gel sample was then smeared on the potassium bromide disk. Next, the disk was positioned in a magnetic holder and scanned from 450–4000 cm^−1^. The sample was scanned 8 times.

### Microbial growth condition and inoculum preparation

*S. mutans* (ATCC 25175) was cultured on brain heart infusion broth (BHI) and incubated at 37 °C for 18–24 h. *P. gingivalis* and *C. albicans* (ATCC 90028) were cultured as previously described [13].

### Transmission electron microscopy (TEM)

The effect of the gels on the ultrastructural morphology of *S. mutans* ATCC 25175, *P. gingivalis* ATCC 33277, and *C. albican*s ATCC 90028 was assessed. The microbes grown in culture media were treated with either 1,000 µg/ml gel or two-fold serial dilution to125 µg/ml. The test bacteria were isolated by centrifuging the cultured cells at 5,000 rpm for 10 min. The cell pellets were used for transmission electron microscopy (TEM) preparation by fixing with 2.5% (v/v) glutaraldehyde, post-fixing with 1% (v/v) osmium tetroxide for 1 h, and dehydrated with ethyl alcohol. The cells were embedded in epoxy resin and serial sections were cut. The sections were mounted on a 300 mesh copper grid, stained with uranyl acetate and lead citrate and examined using an analytical TEM (JEOL JEM-2010 at 200 kV, Japan).

### Inhibition of biofilm formation

The effects of the tooth gels on microbial biofilm formation were tested using the broth microdilution method [19].

### Cell culture conditions

#### Human gingival fibroblast cell line

Human gingival fibroblast (HGF) cell lines were provided by the Faculty of Dentistry, Prince of Songkla University, Thailand. The cell lines were cultured as previously described [20].

### Oral keratinocyte cell line

The oral keratinocyte (OKC) cell line was provided by the Faculty of Dentistry, Chulalongkorn University, Bangkok, Thailand. The cells were cultured as previously described [21]. Briefly, the cell line was grown in Defined Keratinocyte Serum Free Media (Gibco, NY, USA), supplemented with Keratinocyte Growth Factors (Gibco, NY, USA) and 100 U/ml antibiotic–antimycotic (Gibco, NY, USA). The cells were incubated at 37 °C in a 5% CO_2_ atmosphere until reaching 80% confluence, trypsinized with 0.25% trypsin–EDTA (Gibco, NY, USA),and the enzyme activity was inactivated with10% FBS (Gibco, NY, USA) in 1 × PBS. Cell viability was examined using 0.4% trypan blue (Gibco, NY, USA) staining with a light microscope.

### Squamous cell carcinoma cell line

The human tongue squamous cell carcinoma cell line (SCC-25) was purchased from ATCC and cultured as previously described [20].

### Mouse monocyte/ macrophage cell line

A mouse monocyte/macrophage cell line (RAW 264.7, TIB-71™) was obtained from ATCC MD, USA. RAW 264.7 was cultured in Dulbecco’s Modified Eagle Medium (DMEM, Gibco, USA) supplemented with 10% fetal bovine serum (FBS, Gibco, USA), 100 U/ml penicillin/streptomycin (Gibco, USA) as previously described [20].

### Cell viability assay

HGF, OKC, and SCC-25 (1 × 10^5^ cells/ml) were seeded in a 96-well plate and incubated at 37ºC in 5% CO_2_ for 24 h. After incubation, 31.3, 62.5, 125, 250, 500, and 1000 µg/ml samples of the three tooth gels in fresh medium were added into the culture plates. Cells without a sample served as a negative control. After incubation for 24 h, the cell viability (MTT) assay was performed as previously described [20].

### Measurement of nitric oxide production

RAW 267.4 cells (1 × 10^6^ cell/ml) were seeded into 96-well plates in complete medium. After 24 h of incubation, 100 μl of the three tooth gels at 31.1, 62.5, 125, 250 μg/ml, or 1 μg/ml of *E.coli* lipopolysaccharide (LPS, Sigma-Aldrich) as positive control in fresh medium, was added to each well. Wells with media only served as negative controls. The Griess reaction assay was then employed to determine the nitric oxide (NO) content in the cell supernatants as previously described [20].

### In vitro scratch assay

HGF cell line (1 × 10^6^ cells/ well) was seeded into 6-well plates. When the cells were confluent, a linear scratch was generated with a sterile pipette tip in the monolayer. Cell debris was removed by washing three times with 3 ml phosphate buffer saline (PBS) and replaced with 2 ml complete medium containing 250 µg/ml samples of each tooth gel and complete medium without the gel served as the negative control. Images were taken at a 10 × magnification using a light microscope on day 0. The plates were incubated at 37 °C with 5% CO_2_ and images were taken at days 1, 2 and 3. The images acquired for each sample were quantitatively analyzed using computing software (ImageJ, National Institute of Mental Health, Maryland, USA) as previously described [20].

### Microhardness and chemical composition of the enamel surface

The microhardness and chemical composition of the enamel surface before and after brushing with water (control) and the tooth gel with and without fluoride were evaluated. Incisors extracted due to periodontitis were obtained from the Dental Clinic at Thammasat Hospital (ethical approval#IBC 041/2562). Teeth without caries and/or restorations were immersed in a 0.1% thymol solution until used. The teeth were cleaned by removing any calculus and periodontal ligament present on the tooth. The tooth root was cut, and the crown was fixed with self-curing acrylic resin in a 1-inch diameter polyvinyl chloride (PVC) pipe leaving the enamel surface of the tooth 3 mm above the pipe. The enamel surface of each tooth specimen was prepared using a high-speed straight diamond bur to be smooth and parallel with the base of the PVC pipe. An electric toothbrush was used 4 min per time 2 times per day with a 12 h interval for 21 consecutive days. The microhardness of the tooth enamel specimens before and after tooth brushing with the α-MG tooth gels with and without fluoride was measured by Vickers microhardness testing. The microhardness of the specimens were recorded with a microhardness tester (Future tech, FM800, Japan) using a force load and dwell time of 300 gr and 15 s, respectively, at room temperature. The chemical composition of the tooth enamel was investigated using a Fourier Transform spectrometer (FTIR: Thermo Scientific, Nicolet iS5, USA) equipped with a built-in diamond attenuated total reflection (ATR) system. The vibrational spectra of the enamel specimens were measured in the spectral range of 400–4000 cm^−1^ and were recorded at room temperature with 4 cm^−1^ spectral resolution, with 64 scans collected. The surface morphology and element content were examined with a Field Emission Scanning Electron Microscope (FE-SEM: JEOL, JSM7800F, Japan) and Energy Dispersive X-ray Spectroscopy (EDS: Oxford X-Max 20, United Kingdom), respectively. The surface morphology was analyzed via SEM at 2 kV and × 4000 magnification. The elemental analysis was performed with EDS at 15 kV and × 4000 magnification to identify the elemental composition of the enamel surface after brushing. The paired sample T-test was used for statistical analysis.

## Results

### Physical properties of the tooth gels

Three tooth gel formulations with fluoride were prepared; α-MG tooth gel (M), LME tooth gel (L), and α-MG plus LME tooth gel (M + L). The physical properties, pH, and stability of the tooth gels after 6 freeze/thaw cycles were observed and recorded. The three tooth gel formulations were clear with a straw yellow color. The M, L, and M + L tooth gels were initially clear and stayed clear from 1–6 months of storage at room temperature. At accelerated conditions of 45ºC and 4ºC for 6 cycles, which simulated a 2-year shelf life, the tooth gels were clear straw yellow in color. The pH of the formulations was ~ 7 and slightly higher than 7. The α-MG content was similar to that at the initial time point, demonstrating its stability.

### Scanning electron microscopy of the tooth gels

**Fig. 1:**
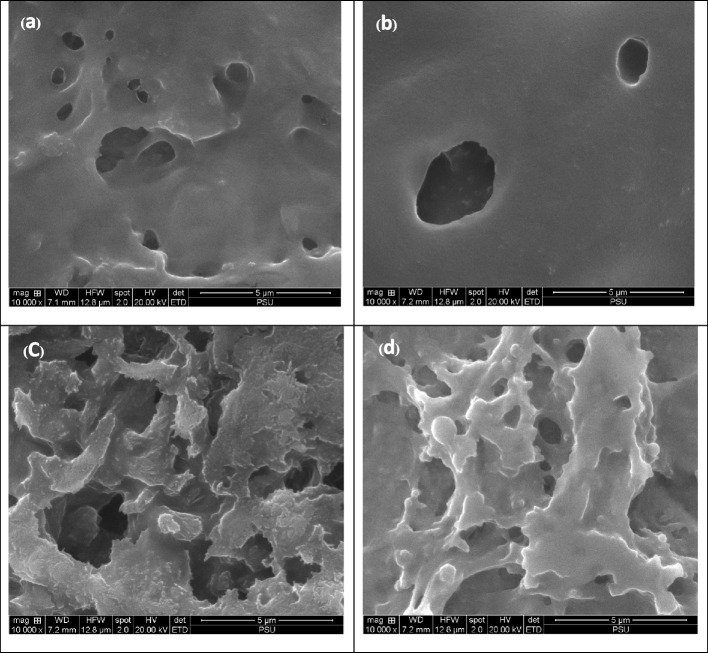
SEM images of tooth gels from blank (**a**), M tooth gel (**b**), L tooth gel (**c**) and M + L tooth gel (**d**)

The scanning electron microscopic images of the three tooth gel formulations are seen in Fig. 1. The blank tooth gel had a relatively less irregular surface. The M tooth gel presented the smoothest surface with some holes on the surface. In contrast, the L tooth gel had a rough surface with porous structures. Thus, it is expected to have high porosity. The M + L tooth gel had a rough surface with fewer holes compared with the L tooth gel.

###  Fourier transform-infrared spectroscopy of the tooth gels

**Fig. 2:**
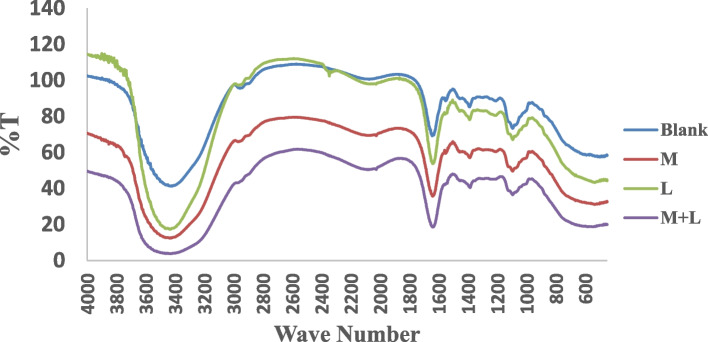
FT-IR of tooth gels from blank, M tooth gel (M), L tooth gel (L) and M + L tooth gel (M + L)

 Fourier transform-infrared spectroscopy of the three formulations of the tooth gel is shown in Fig. 2. The FT-IR spectra of the tooth gels revealed that the gel contained a large amount of water as indicated by a large broad band of OH stretching from 3200–3600 cm^−1^. The xylitol and poloxamer 407 peaks were overwhelmed by the broad band of water. Typically, the peaks of xylitol bound by OH stretching were observed at 3354 cm^−1^ and 3284 cm^−1^ and intense CH peaks at 1418 cm^−1^. The poloxamer 188 was characterized by main absorption peaks at 3437, 2883, 1344, and 1111 cm ^−1^, which can be attributed to OH stretching, aliphatic C-H stretch, in-plane O-H bend, and C-O stretch, respectively. Sharp peaks were found at 1650 cm^−1^, which is carbonyl stretching found in all tooth gel formulations. This was contributed from the Pemulen TR-2 and perhaps the α-MG and LME. Other peaks were observed at 1100 cm^−1^and 1400 cm^−1^.

### Antimicrobial activities of the tooth gels

**Fig. 3:**
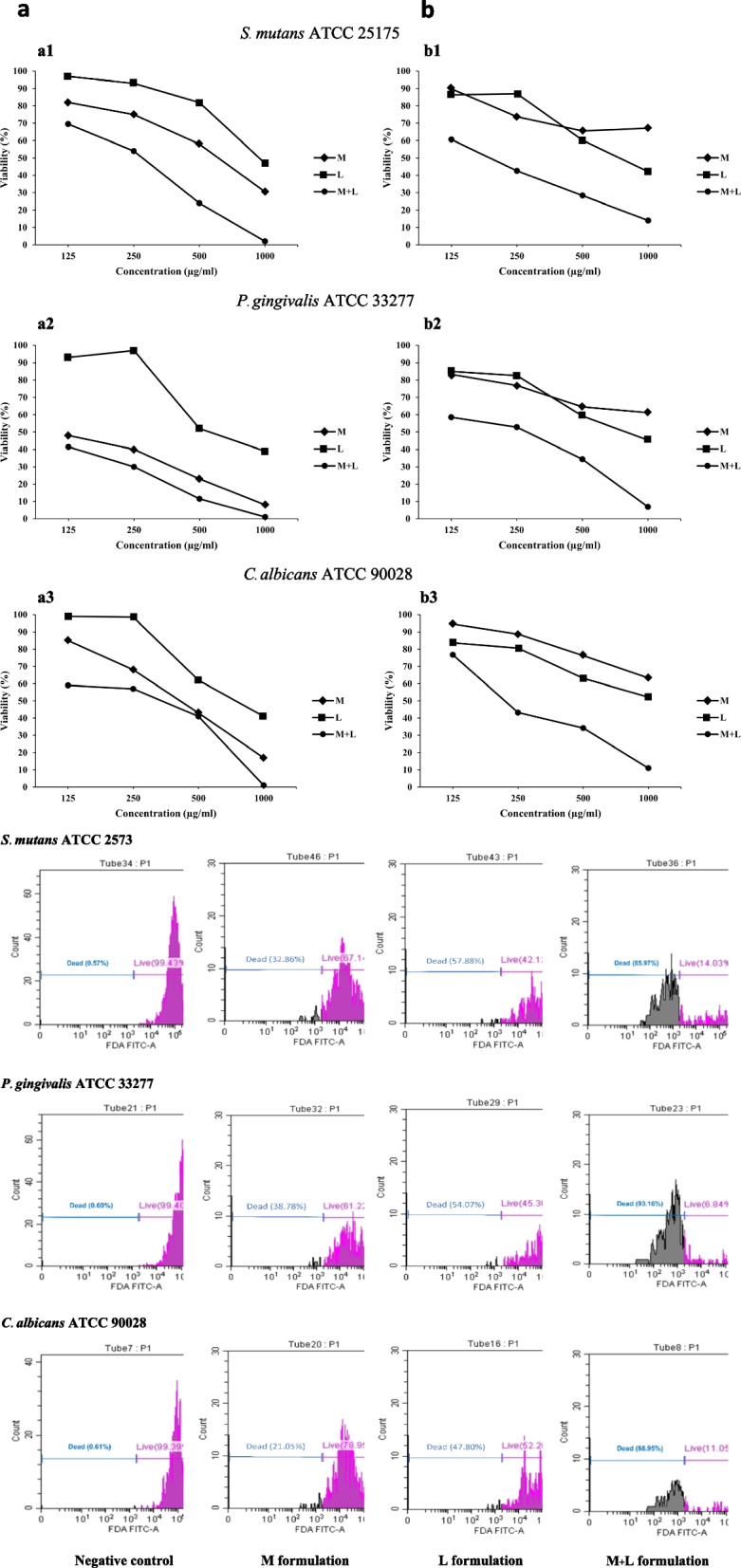
%Viability of the pathogens after being incubated with the 1 mg/ml tooth gel formulations using flow cytometry. The viability of the microbes after incubation with four serial dilutions of the 1% tooth gel for 18 h significantly decreased when the α-MG or LME concentration increased. The α-MG had a lower antimicrobial activity than that of LME. Synergy between the two compounds were noted when they were combined at only half the concentration of each component (M = α-MG tooth gel; L = LME tooth gel; M + L = α-MG and LME tooth gel)

The viability of *S. mutans* ATCC 25175, *P. gingivalis* ATCC 33277, and *C. albicans* ATCC 90028 after incubation with formulations is seen in Table S1-S3. The viability and the flow cytometric analyses revealed the formulations’ antimicrobial activity (Fig. 3). The results indicated that % viability of the control was 99.43, 99.40, and 99.39 for *S. mutans*, *P. gingivalis*, and *C. albicans*, respectively. The *S.mutans* viability after incubation with four serial dilutions of the tooth gel for 18 h significantly decreased when the α-MG or LME concentration increased. The α-MG had a lower antibacterial activity than that of LME. However, when these two compounds were combined at only half the concentration of each component, the antibacterial activity significantly increased. For anaerobic *P. gingivalis,* the α-MG activity was lower than that of the LME compound. In contrast, when they were combined, the activity significantly increased. The results also indicated that the α-MG and LME compounds enhanced the antifungal activity against *C. albican*s. The individual activity of each compound was less than that of their combination.

**Fig. 4:**
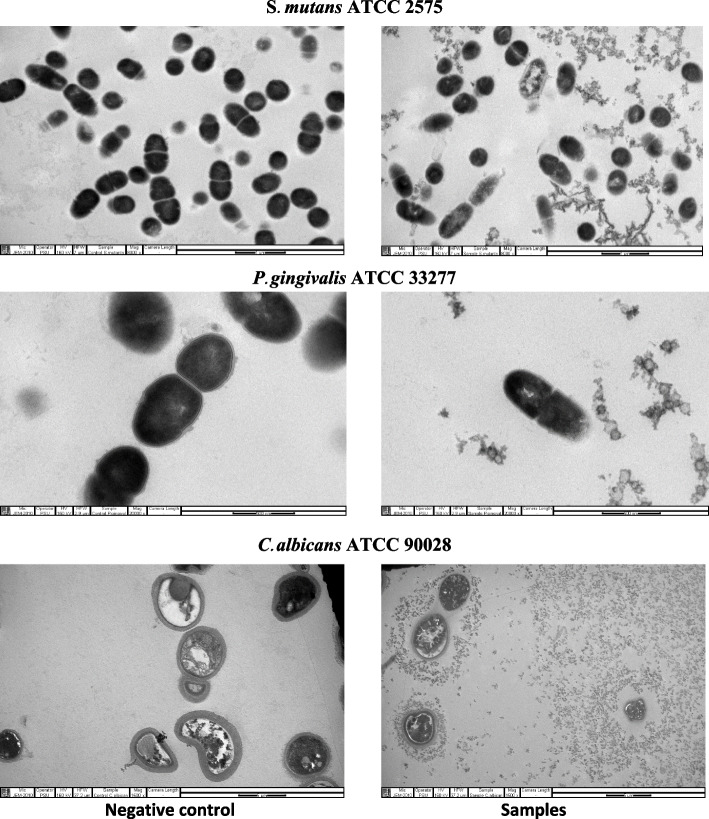
Transmission electron micrographs of *S. mutans* ATCC 2575*, P. gingivalis* ATCC 33277, and *C. albicans* ATCC 90028 after incubation with the 1 mg/ml M + L tooth gel formulation

After the bacteria and fungus were challenged with the tooth gel containing M + L, the morphology of the microbes was observed with TEM (Fig. 4). The untreated *S. mutans* cells were round or oval and some bacterial cells were seen to be dividing into two cells. When *S. mutans* was treated with the M + L samples, the cell morphology changed and cell degradation was observed. Some cells were lysed with internal clumping. In contrast, the untreated *P.gingivalis* cells were round or oval and some cells were dividing. When the cells were treated with M + L tooth gel, one of the two dividing cells appeared to be lysed. With the *C. albicans* yeast cells, the untreated cells were round, oval, and bean-shaped. When the cells were treated with the M + L tooth gel, the yeast cells were degraded with segregation.

### Antibiofilm formation effect of the tooth gels

**Fig. 5:**
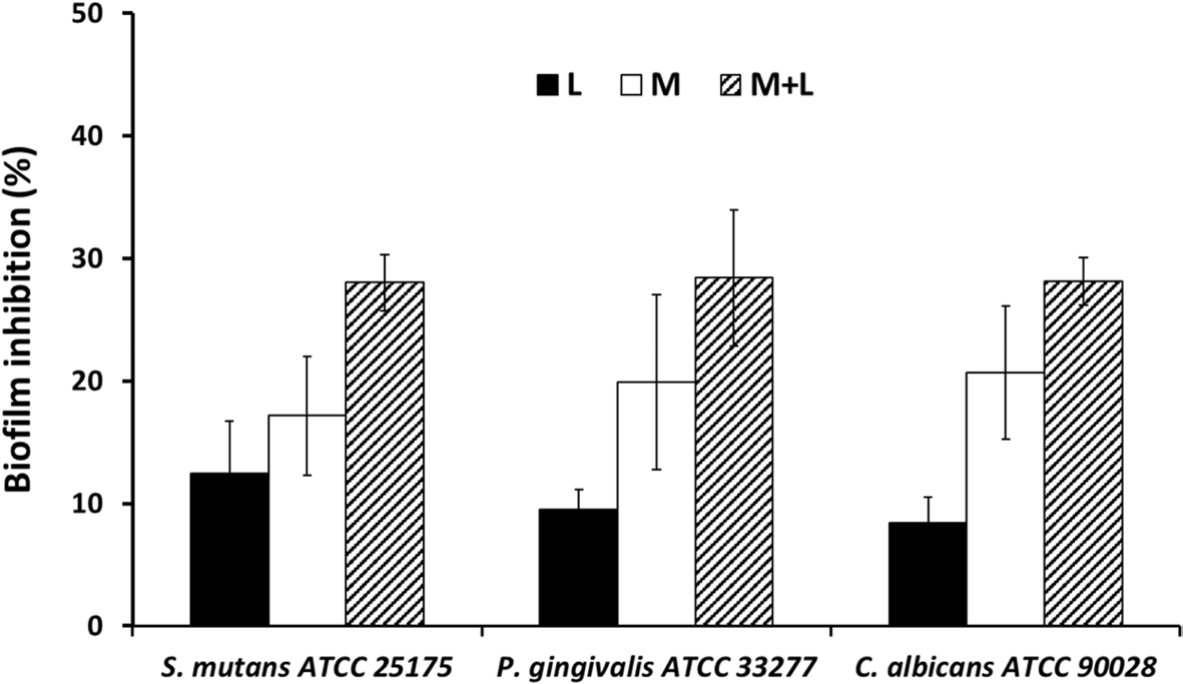
Antibiofilm formation of the tooth gels α-MG (M) and LME (L) synergistically increased the tooth gel’s antibiofilm formation activity against *S. mutans*, *P. gingivalis,* and *C. albicans* by up to 30%

α-MG and LME synergistically increased the tooth gel’s antibiofilm formation activity against *S. mutans*, *P. gingivalis,* and *C. albicans* by up to 30% (Fig. 5).

### Effects of the tooth gels on cell viability

The effects of the tooth gels on HGF, OKC, and oral cancer cell line SCC-25 cell viability were assessed using the MTT assay. Three tooth gel formulations were not toxic to fibroblasts at 250 µg/ml. Cell viability of ~ 80% was observed for the M + L group at 500 µg/ml (Figure S1a). The M + L tooth gel was not toxic to oral keratinocytes up to 0.63 mg/ml (Figure S1b), however, toxicity was observed at 1.25 mg/ml. In addition, the tooth gel reduced SCC-25 cell viability at 50 mg/ml (Figure S1c).

### Anti-inflammatory activity of the tooth gels

The percent inhibition of NO was determined to assess the anti-inflammatory activity of the tooth gels. The anti-inflammatory activity of the tooth gels was evaluated using RAW 264.7 cells. However, the tooth gels did not demonstrate NO inhibition (Figure S2).

### Wound healing effects of the tooth gels

To evaluate the wound healing effects of the tooth gels, cell migration was measured using an in vitro scratch assay. The confluent HGF cell layer was scratched and incubated with the tooth gels (1 mg/ml) for 3 days. The effect of the tooth gel on in vitro HGF migration is displayed in Table 1 (*n* = 3). The results indicated that cell migration did not significantly change in the tooth gel-treatment groups compared with the control group (Figure S3).

**Table 1:** Effect of the tooth gel formulations in the in vitro scratch assay using gingival fibroblasts, (*n* = 3)

Day	%Migration rate cells (Mean ± s.d.)			
	M	L	M + L	Negative control
0	0.00 ± 0.0	0.00 ± 0.0	0.00 ± 0.0	0.00 ± 0.0
1	20.77 ± 1.88	19.10 ± 0.29	19.17 ± 1.83	19.83 ± 1.77
2	72.70 ± 1.0	72.75 ± 1.69	72.10 ± 1.83	70.30 ± 3.19
3	97.21 ± 0.40	93.59 ± 1.40	98.88 ± 1.04	99.92 ± 0.62

### Effects of the tooth gels on enamel microhardness and chemical composition

**Fig. 6:**
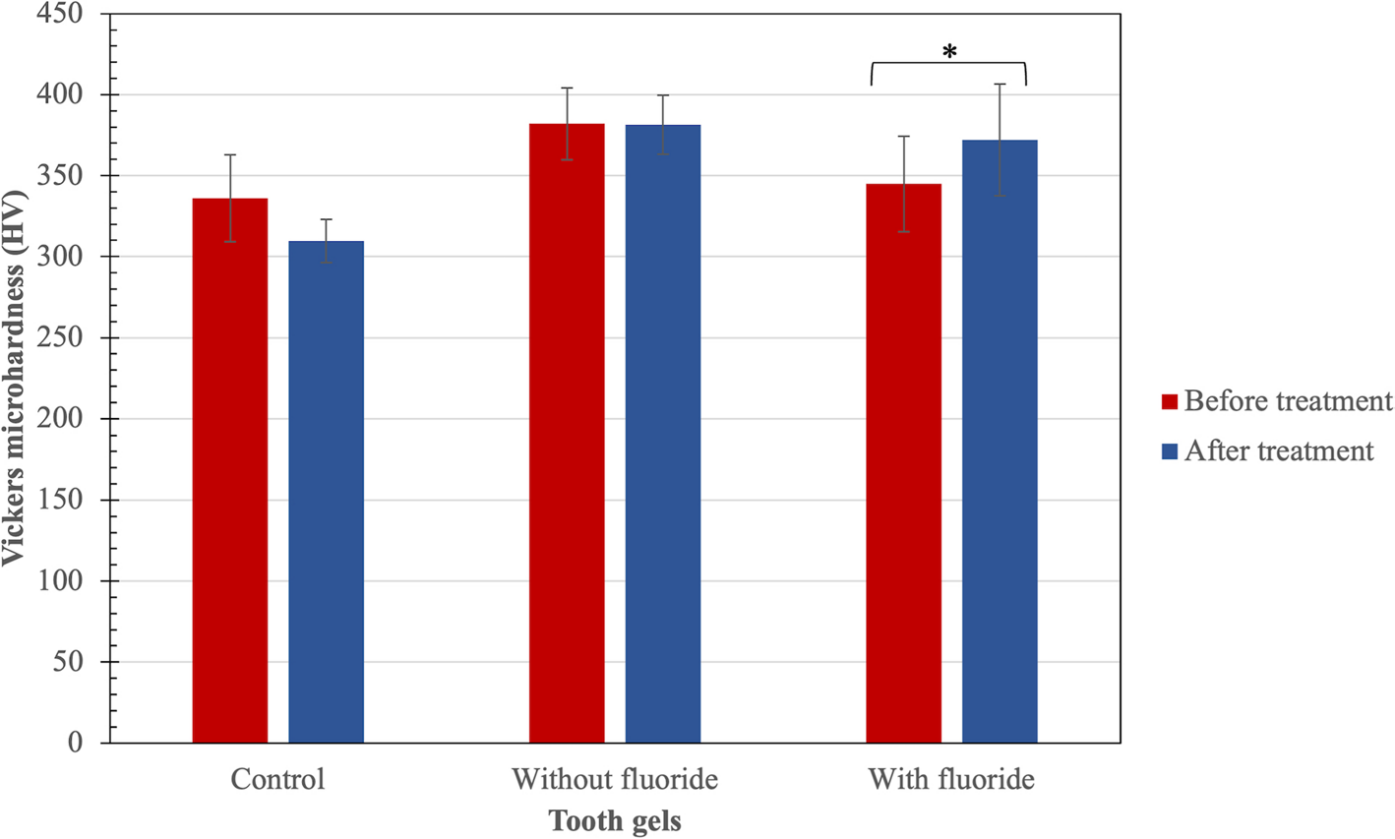
Microhardness of the enamel surface before and after brushing with the different tooth gels The enamel microhardness and chemical composition were determined before and after brushing with the tooth gels. The tooth gel without fluoride and the control did not affect the enamel microhardness. In contrast, the microhardness after brushing with the tooth gel plus fluoride was higher than that before brushing

The enamel microhardness and chemical composition were determined before and after brushing with the tooth gels. The control tooth gel without fluoride did not affect the enamel microhardness. In contrast, the microhardness after brushing with the tooth gel plus fluoride was higher than that before brushing (Fig. 6).

**Fig. 7:**
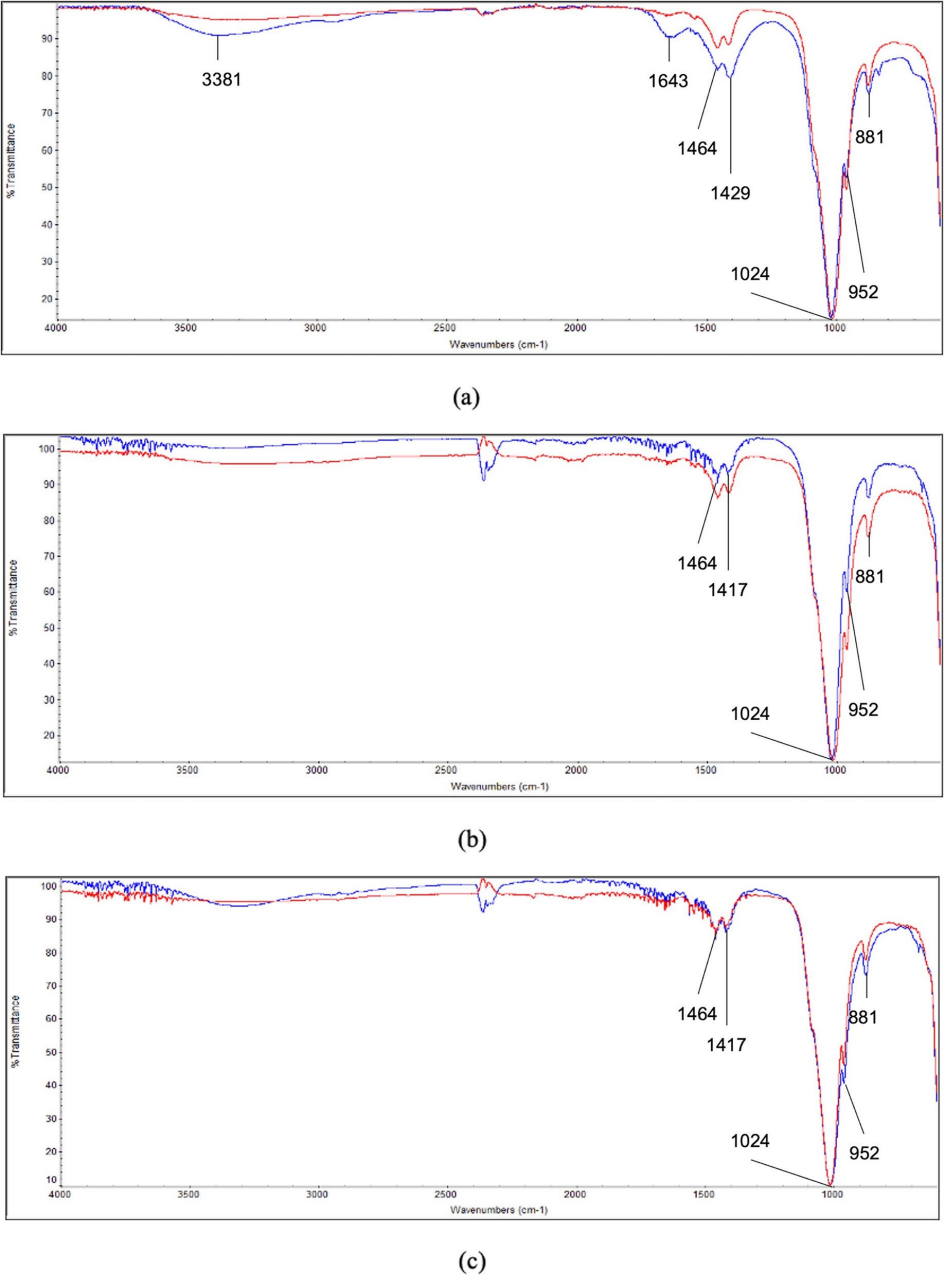
Fourier-transform infrared (FT-IR) spectra of before (red line) and after (blue line) brushing with different tooth gels: (**a**) water (control), **b** tooth gel without fluoride, and **c** tooth gel with fluoride. FT-IR spectra of enamel surface before and after tooth brushing with water, tooth gel with and without fluoride revealed the ranges corresponding to the CO_3_^2−^ region (890–850 cm^−1^ and 1450–1350 cm^−1^) and PO_4_^3−^ region (1230–959 cm^−1^). The hydroxyl (OH) group transmittance peak was recorded between 3600 and 2800 cm^−1^ associated with OH^−^ (1630 cm^−1^) for the enamel surface after brushing with water

The FT-IR spectra of the enamel surface before and after brushing with water, and the tooth gels with and without fluoride revealed the peak ranges corresponding to the CO_3_^2−^ region (890–850 cm^−1^ and 1450–1350 cm^−1^) and PO_4_^3−^ region (1230–959 cm^−1^) (Fig. 7). Furthermore, the hydroxyl (OH) group transmittance peak was recorded between 3600 and 2800 cm^−1^ associated with OH^−^ (1630 cm^−1^) for the enamel surface after brushing with water.

**Fig. 8:**
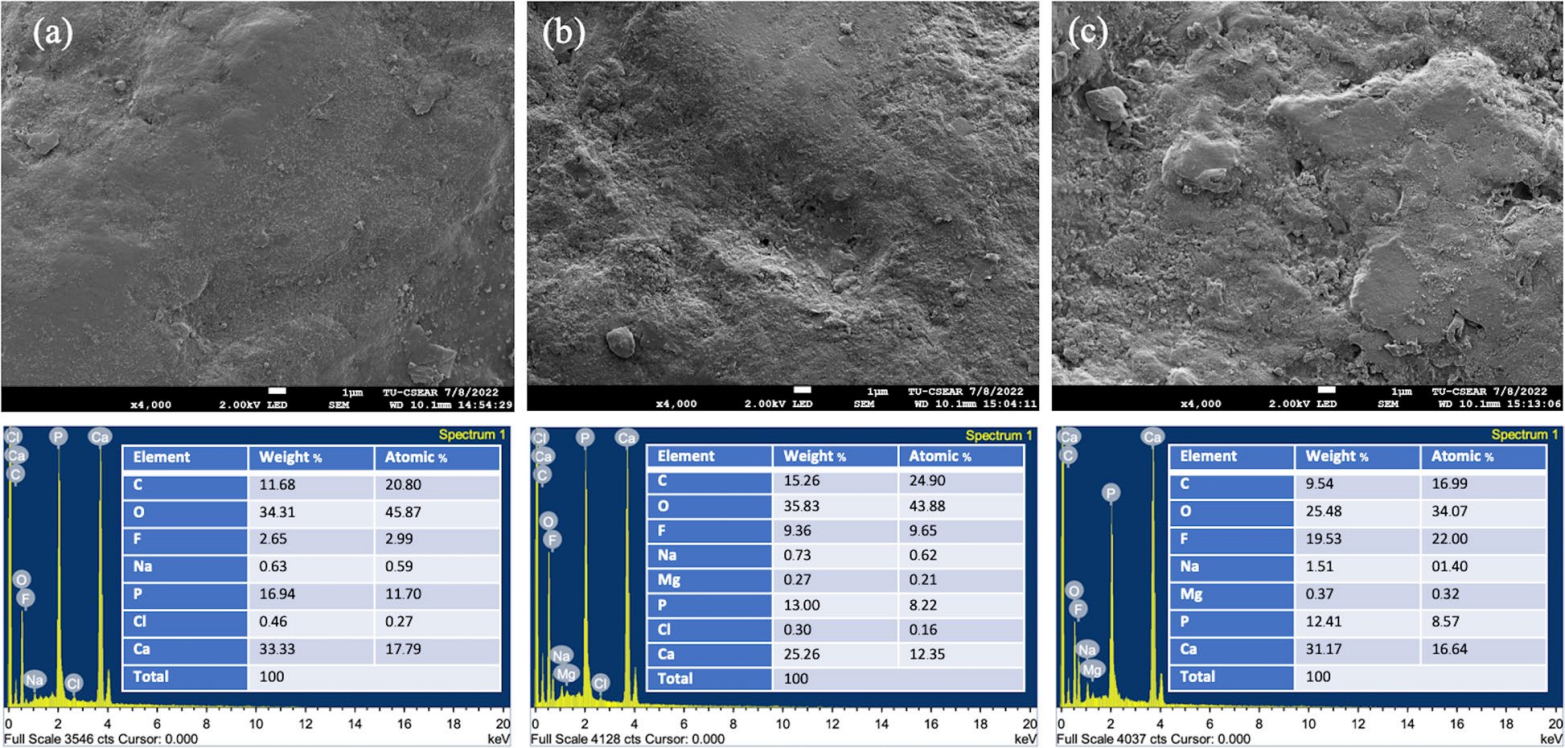
Upper panel shows SEM micrographs of the enamel surface after brushing with the different tooth gels at 4000x: (**a**) water (control), **b** tooth gel without fluoride, and **c** tooth gel with fluoride. The SEM micrographs revealed that the tooth enamel surface was rough, likely due to the mechanical action of the brushing. The enamel surface after brushing with the tooth gel without fluoride presented a roughness similar to that of the control. However, the enamel surface after brushing with the tooth gel was covered with different size and number of deposits, which was evident that brushing with the tooth gel without fluoride generated fewer deposits than brushing with the tooth gel plus fluoride. Lower panel shows EDS analysis of the enamel surface after brushing with the different tooth gels: (**a**) water (control), **b** tooth gel without fluoride, and **c** tooth gel with fluoride. The results illustrated that the tooth enamel sample brushed with the tooth gel plus fluoride has a large amount of fluoride and Ca/P ratio. The control sample brushed with water possesses the lowest F weight percentage, and similar Ca/P ratio with enamel sample brushed with the tooth gel without fluoride

The SEM micrographs revealed that the tooth enamel surface was rough, which was likely caused by the mechanical action of the toothbrush (Fig. 8 upper panel). The enamel surface after brushing with the tooth gel had a roughness similar to that of the control. However, the enamel surface after brushing with the tooth gel was covered with different sizes and numbers of deposits, indicating that brushing with the tooth gel without fluoride generated fewer deposits than brushing with the tooth gel plus fluoride (Fig. 8 upper panel).

The EDS analysis revealed that the tooth enamel specimen brushed with the tooth gel plus fluoride had a high amount of fluoride and Ca/P ratio. The control sample brushed with water possessed the lowest F weight percentage, and a similar Ca/P ratio with the enamel specimens brushed with the tooth gel without fluoride (Fig. 8 lower panel).

## Discussion

This study demonstrated that the α-MG and LME in the tooth gel had synergistic effects against *S. mutans, P. gingivalis, C. albicans,* and their biofilm formation. The tooth gels were demonstrated to be stable because the accelerated conditions can predict the long term stability of gels, ointments, and creams. In this case, the 6-cycles stability assay of the tooth gels indicated that the product would be stable for longer than 2 years. In addition to the physical stability of the tooth gels, the α-MG in the formulation remained chemically stable. The three tooth gel formulations resulted in different surface and porosity that may be due to the different active ingredients in each gel. This may lead to different interactions and adhesions on the teeth and buccal surface.

The tooth gels were not cytotoxic to oral cells at the therapeutic dose. Moreover, at higher concentrations the M + L tooth gel inhibited oral cancer cell proliferation. The M + L tooth gel also affected the morphology of those three microbes. The incorporation of fluoride into the tooth gel increased the microhardness of the tooth enamel.

In the present study, the flow cytometric analysis revealed the synergistic effects of the a-MG and LME in the tooth gel against *S. mutans, P. gingivalis,* and* C. albicans*. The data revealed that the viability of *S. mutans* after incubation with the tooth gel significantly decreased when the concentration of α-MG or LME increased. α-MG had a lower antibacterial activity than that of LME. However, when these two compounds were combined the antibacterial activity was significantly increased. These findings confirmed that the two compounds synergized each other. For anaerobic *P. gingivalis,* α-MG activity was lower than that of the LME compound. In contrast, when they were combined, the activity was significantly enhanced. The results also indicated that α-MG and LME promoted the antifungal activity against *C. albican*s. The synergistic effects on the antifungal activity of the two compounds were found because the individual activity was less that of the combination. In addition, synergistic effects of α-MG and LME were detected on antibiofilm formation by *S. mutans*, *P.gingivalis,* and *C. albicans* when the microbes were challenged with the tooth gel containing α-MG plus LME.

The synergistic effects of α-MG and LME may be explained by the fact that α-MG is a multi-target inhibitor of the microbes. The antimicrobial activity of α-MG is due to potently inhibiting acid production by the microbes and the membrane enzymes. α-MG also inhibited the glycolytic enzymes aldolase, glyceraldehyde-3-phosphate dehydrogenase, and lactic dehydrogenase. Glycolysis of the microbes in biofilms is also inhibited by α-MG [22]. Other targets for inhibition by α-MG include (i) malolactic fermentation, and (ii) nicotinamide adenine dinucleotide phosphate **(**NADH) oxidase, the major respiratory enzyme for *S. mutans* [23]. A previous study reported that oral spray containing α-MG and LME show potent antimicrobial activity against *S. mutans*, *P. gingivalis*, and *C. albicans* [10]. A recent study demonstrated that LME and Artocarpin-rich extract (ARE) as well as its combinations with ampicillin altered the membrane permeability of Methicillin-resistant *Staphylococcus aureus *(MRSA) resulting in the leakage of the intracellular materials. Each compound also inhibited the biofilm formation of microbes [24]. Thus, our results were consistent with the previous findings that the combination of α-MG and LME in the formulation enhances the antimicrobial and antibiofilm formation effects in vitro.

The present study also revealed that the tooth gels affected the morphology of the three microbes tested. When the bacteria and fungus were challenged with the tooth gel containing α-MG plus LME, changes in microbe morphology were observed using TEM. The untreated *S. mutans* and *P. gingivalis* cells were round or oval and some bacterial cells were dividing into two cells. In contrast, when *S. mutans* and *P. gingivalis* cells were treated with the M + L samples the bacterial cell morphology changed with cell degradation observed. Some *S. mutans* cells were lysed with clumping internally and the one side of the *P. gingivalis* cell membrane was degraded. In addition, the untreated *C. albicans* yeast cells were round, oval, or bean-shaped. When the cells were treated with M + L tooth gel, the yeast cells were degraded with segregation. These findings confirmed that the tooth gel containing α-MG plus LME possessed antimicrobial activity against *S. mutans, P. gingivalis,* and* C. albicans*.

α-MG has been used in a topical gel to treat chronic periodontitis [15, 16], and as an antibacterial component in adhesive paste to prevent dental caries [14]. However, there is no report on adding fluoride in the formulations containing α-MG. It should be noted that fluoride in the tooth gel formulations is strongly antimicrobial. The fluoride effect may contribute to the anticaries activity by reducing the metabolism and growth of the microbes [25]. The effect of fluoride on bacterial metabolism might be due to inducing cytoplasmic acidification and glycolytic enzyme inhibition. Fluoride can affect the metabolism of bacteria through different mechanisms. It can act directly by inhibiting enzymes, such as the glycolytic enzyme enolase. The actions of fluoride on reducing the cariogenicity of dental plaque are related to its weak-acid character. Fluoride enhances membrane permeability to protons and compromises the function of F-ATPases in exporting protons, thereby inducing cytoplasmic acidification and acid inhibition of glycolytic enzymes. Fluoride also reduces the acid tolerance of the microbes. It is most effective at an acid pH. In the acidic conditions of cariogenic plaque, 0.1 mM fluoride causes complete arrest of glycolysis by intact *S.mutans* cells [26].

In the present study, the three tooth gel formulations did not demonstrate anti-inflammatory activity and did not promote wound healing. However, previous studies revealed that in addition to killing oral pathogens, α-MG also mediated the anti-inflammatory response by lowering the expression of nuclear factor kβ (NfkB) and receptor activator of nuclear factor kβ ligand [10, 23] reported that an oral spray containing α-MG had an anti-inflammatory effect and Tangsuksan et al. [20] demonstrated that a mucoadhesive film containing α-MG enhanced wound healing. The inconsistent findings of the present study may be due to the different ingredients in the formulations that may affect the release of the active ingredient of α-MG.

The Vickers microhardness test is commonly used to evaluate the surface hardness of enamel [27–29]. Prior studies have investigated using natural or herbal-based toothpaste to improve the hardness of the tooth enamel surface. It was found that toothpaste containing a natural or herbal ingredient, such as theobromine, increased the enamel surface hardness [30]. The interstitial reactions between the hydroxyapatite (HA) crystals and theobromine on the enamel surface explained these results. There is also a report that toothpaste containing green tea extract increased the hardness of the demineralized dentin [31]. The authors suggested that the increased enamel hardness was due to the carbonated-hydroxyapatite crystals that formed and covered the demineralized area. In contrast, the results of our study indicated that there was no significant difference in the microhardness of the enamel surface between before and after brushing with the tooth gel. This may be because the pH of the tooth gel was ~ 7 (more than 5.2, the critical pH for enamel), which cannot demineralize the enamel surface [32], and that the enamel surface hardness is not decreased. The unchanged enamel surface hardness suggests that the compounds in the tooth gel may not play a role in tooth enamel remineralization. However, adding fluoride into the tooth gel significantly increased the enamel surface hardness. This is due to the incorporation of fluoride into HA, forming fluorohydroxyapatite crystals.

FT-IR spectroscopy is used to analyze the changes in the chemical structure and composition of the tooth enamel surface [33, 34]. The peaks in these spectra were assigned according to the literature [35, 36]. In the present study, there were no changes in the enamel spectra after brushing with either tooth gel. Interestingly, after brushing with water, the enamel spectra had strong transmittance bands between 3600 and 2800 cm^−1^, and at 1630 cm^−1^ associated with water bands, which may be because enamel is permeable to water [37, 38]. In contrast, after brushing with the tooth gel plus fluoride, the enamel surface was covered with the compounds and fluorohydroxyapatite crystals, as previously discussed. Consequently, the water bands are not present in the enamel FTIR spectra after brushing with either tooth gel.

SEM–EDS has been used for tooth enamel surface analysis [33, 34]. SEM images demonstrate the surface morphology, and EDS reveals the chemical composition of the tooth enamel sample in the selected micro-area of its surface. In this study, the SEM micrographs of the enamel surface after brushing are in line with the microhardness results. It should be noted that the deposit-covered surface is a sign of remineralization [39]. The tooth gel with fluoride may form fluorohydroxyapatite, which fills in the irregularities of the rough enamel surfaces, leading to increased microhardness. The EDS results illustrated that fluoride, calcium, and phosphorus are the main elements of fluorohydroxyapatite, therefore, the F weight percentage and Ca/P ratio were monitored in this research. It was found that the fluoride weight percentage and Ca/P ratio were highest for the enamel surface brushed with the tooth gel plus fluoride, which support the explanation as discussed earlier.

Dental caries and periodontal diseases are the two most common oral diseases worldwide. The misuse and overuse of antibiotics may lead to the development of antimicrobial resistance. In addition to medicinal plants with antimicrobial activity and antibiofim formation effects, efforts have also been made towards using antimicrobial peptides (AMP), which are known as host defense peptides [40]. The amino acid composition of AMPs can be modified for broad spectrum application and to target specific pathogens [41]. A recent study by Yang et al. [42] revealed that the modified peptide 1018, a novel hydroxyapatite-binding AMP, had high antimicrobial activity against biofilm microbes and reduced biofilm volume and may be considered as a promising agent for use in oral antibiofilm strategies in the future.

In conclusion, the present study demonstrated that the α-MG and LME in the tooth gel synergistically increased its antimicrobial activity against *S. mutans, P. gingivalis, and C. albicans* and antibiofilm formation effect. The fluoride tooth gel containing α-MG plus LME affected the morphology of these microbes. The tooth gel was not toxic to normal oral cells at a therapeutic dose. At higher concentration, however, the tooth gel inhibited the oral cancer cell line’s proliferation. The incorporation of fluoride into the tooth gel increased the microhardness of the tooth enamel. The limitation of this study was that it was performed in vitro. Clinical trials in patients brushing with this tooth gel should be performed to confirm the findings.

### Supplementary Information

**Additional file 1:  Table S1. **%Viability of *S. mutans* ATCC 25175 after being incubated with the formulations. **Table S2.** %Viability of *P. gingivalis *ATCC 33277 after incubated with formulation. **Table S3.** %Viability of *C.* *albicans *ATCC 90028 after incubated with formulation. **Figure S1.** Cytotoxicity of the tooth gels. **Figure S2.** Amount of NO produced by the RAW cells in response to the tooth gels (mean±s.d., *n*=3). **Figure S3.** Migration of the gingival fibroblast cells after being incubated with the tooth gel formulations at various time points (mean±s.d., *n*=3).

## Data Availability

The data that support the findings of this study are available from Thammasat University, but restrictions apply to the availability of these data, which were used under license for the current study, and so are not publicly available. Data are however available from the corresponding author upon reasonable request and with permission of Thammasat University.

## Abbreviations

α-MG: α-Mangostin
AMP: Antimicrobial peptides
ATR: Attenuated total reflection
BHI: Brain heart infusion broth
EDS: Energy Dispersive X-ray Spectroscopy
FE-SEM: Field Emission Scanning Electron Microscope
FT-IR: Fourier transform-infrared spectroscopy
HA: Hydroxyapatite
LME: Lawsone methyl ether
LPS: Lipopolysaccharide
MRSA: Methicillin-resistant *Staphylococcus aureus*
MTT: Methylthiazol tetrazolium
NADH: Nicotinamide adenine dinucleotide phosphate
HGF: Human gingival fibroblast
NfkB: Nuclear factor kβ
NO: Nitric oxide
OKC: Oral keratinocyte
PBS: Phosphate buffer saline
PVC: Polyvinyl chloride
SCC-25: Squamous cell carcinoma cell line-25
SEM: Scanning Electron Microscopy
TEM: Transmission Electron Microscopy
