# Supplementary material for: α-Mangostin and lawsone methyl ether in tooth gel synergistically increase its antimicrobial and antibiofilm formation effects in vitro
Source: BMC Oral Health. 2023 Nov 8;23:840. doi: 10.1186/s12903-023-03511-z (PMC10631194; doi:10.1186/s12903-023-03511-z)
Supplement: Supplementary file 1 — Additional file 1: Table S1. %Viability of S. mutans ATCC 25175 after being incubated with the formulations. Table S2. %Viability of P. gingivalis ATCC 33277 after incubated with formulation. Table S3. %Viability of C. albicans ATCC 90028 after incubated with formulation. Figure S1. Cytotoxicity of the tooth gels. Figure S2. Amount of NO produced by the RAW cells in response to the tooth gels (mean±s.d., n=3). Figure S3. Migration of the gingival fibroblast cells after being incubated with the tooth gel formulations at various time points (mean±s.d., n=3). [file 12903_2023_3511_MOESM1_ESM.docx]

**Table S1.** %Viability of *S.mutans* ATCC 25175 after being incubated with the formulations.

| **Samples** | **Concentration (µg/ml)** | | | |
| --- | --- | --- | --- | --- |
|  | **125** | **250** | **500** | **1,000** |
| **M** | 89.97**±**0.88 | 73.71**±**0.75 | 65.54**±**0.45 | 67.14**±**0.45 |
| **L** | 86.40**±**0.39 | 87.03**±**0.35 | 60.22**±**0.20 | 42.12**±**0.55 |
| **M+L** | 60.59**±**0.56 | 42.58**±**0.55 | 28.34**±**0.45 | 14.03**±**0.25 |
| **Control** | **99.43±0.21** | | | |

**Table S2.** %Viability of *P. gingivalis* ATCC 33277 after incubated with formulation.

| **Samples** | **Concentration (µg/ml)** | | | |
| --- | --- | --- | --- | --- |
|  | **125** | **250** | **500** | **1,000** |
| **M** | 83.33**±**0.45 | 76.89**±**0.95 | 64.56**±**0.65 | 61.22**±**0.35 |
| **L** | 85.04**±**0.40 | 82.65**±**0.75 | 59.28**±**0.75 | 45.30**±**0.44 |
| **M+L** | 58.55**±**0.60 | 52.8**±**0.90 | 34.43**±**0.55 | 6.84**±**0.65 |
| **Control** | **99.40±0.22** | | | |

**Table S3**. %Viability of *C. albican* ATCC 90028 after incubated with formulation.

| **Samples** | **Concentration (µg/ml)** | | | |
| --- | --- | --- | --- | --- |
|  | **125** | **250** | **500** | **1,000** |
| **M** | 94.58**±**0.55 | 88.78**±**0.85 | 76.27**±**0.35 | 63.19**±**0.35 |
| **L** | 83.68**±**0.75 | 80.60**±**0.80 | 63.08**±**0.75 | 52.20**±**0.25 |
| **M+L** | 76.74**±**0.85 | 43.24**±**0.55 | 34.22**±**0.45 | 11.05**±**0.25 |
|  | **99.39±0.22** | | | |

**a**

**
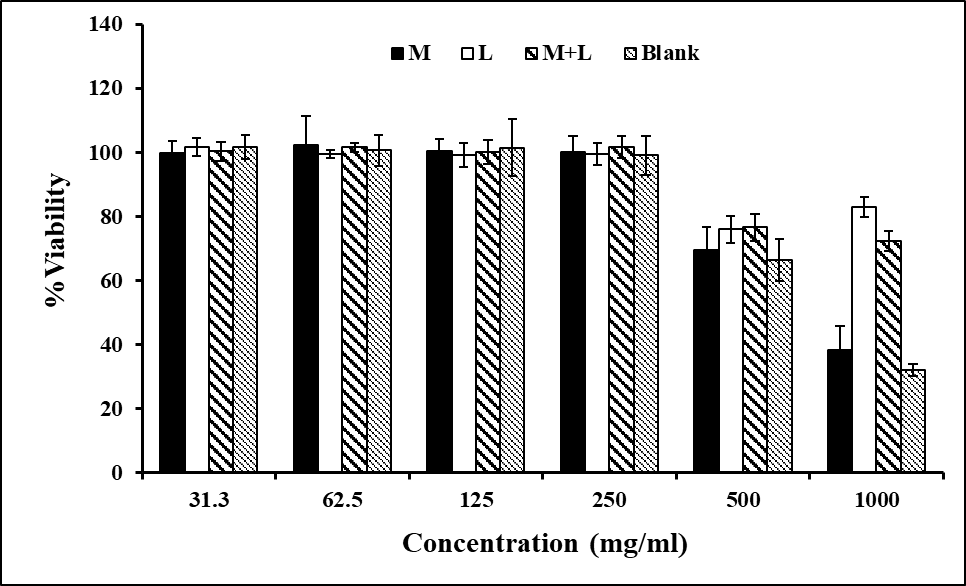
**

**b c**

**Figure S1** Cytotoxicity of the tooth gels

Human gingival fibroblasts, oral keratinocytes, and SCC-25 cell line were treated with the samples at various concentrations (mean ± s.d., n = 4). The three tooth gel formulations at the concentration up to 250 µg/ml demonstrated no toxicity to fibroblasts and at the concentration of 500 µg/ml the cell viability of ~80% was observed (a). The M+L tooth gel was not toxic to oral keratinocytes up to 0.63 mg/ml, but was toxic at 1.25 mg/ml (b). In addition, the tooth gel reduced the SCC-25 cell viability at 50 mg/ml (c). (M=α-MG tooth gel; L= LME tooth gel; M + L= α-MG and LME tooth gel)


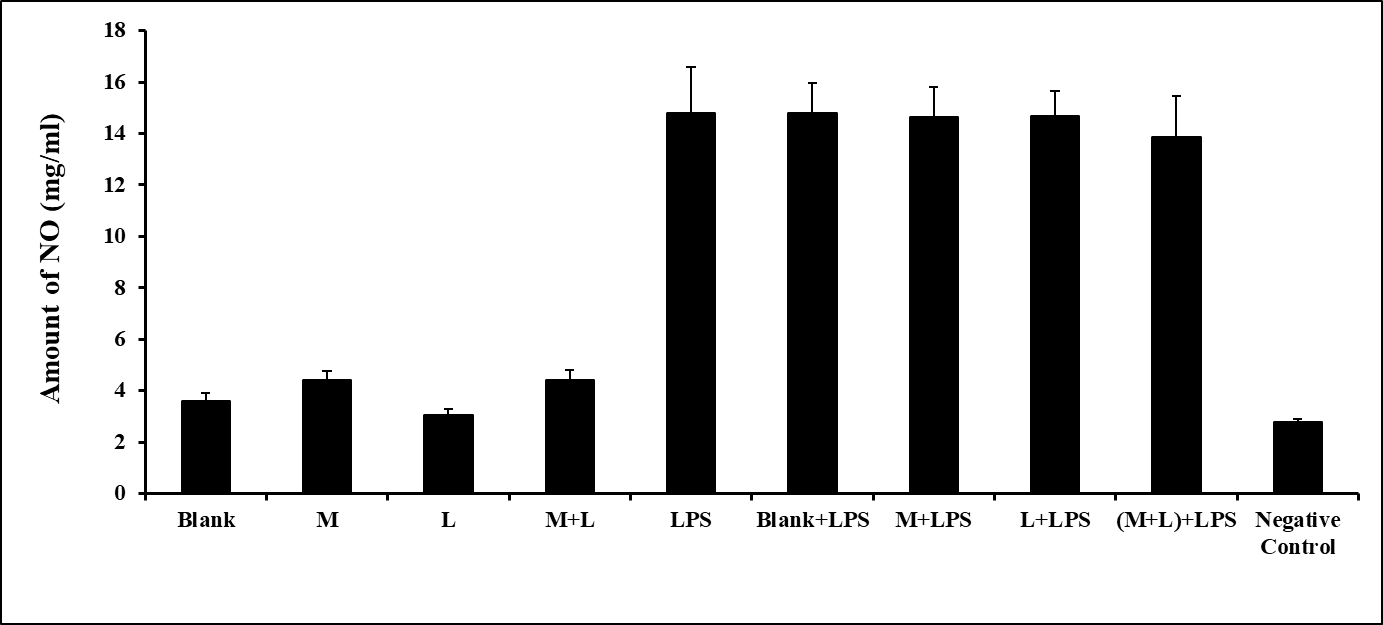


**Figure S2** Amount of NO produced by the RAW cells in response to the tooth gels (mean±s.d., n=3).

The tooth gels did not demonstrate NO inhibition, implying that there was no anti-inflammatory effect. (M=α-MG tooth gel; L= LME tooth gel; M + L= α-MG and LME tooth gel; LPS= lipopolysaccharide)

|  | **Negative control** | **Formulation M** | **Formulation L** | **Formulation M+L** |
| --- | --- | --- | --- | --- |
| **Day 0** | 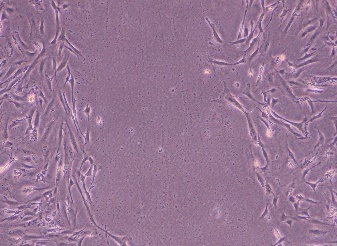 | 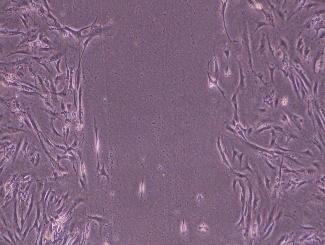 | 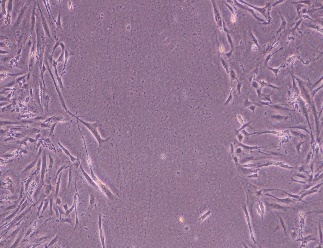 | 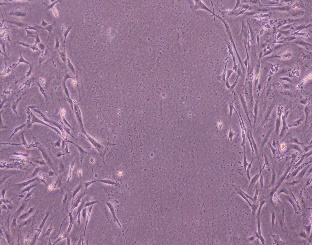 |
|  |  |  |  |  |
| **Day 1** | 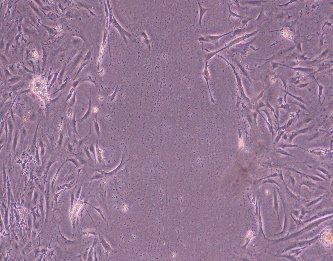 | 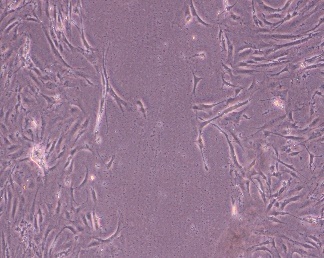 | 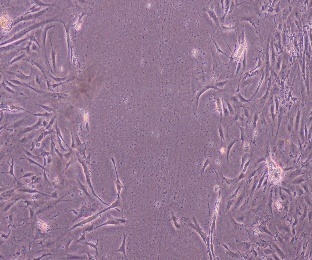 | 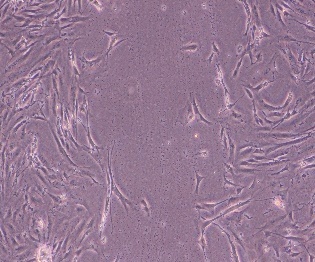 |
|  |  |  |  |  |
| **Day 2** | 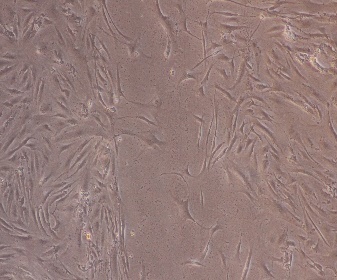 | 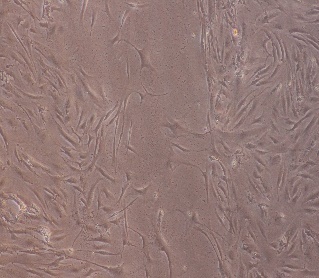 | 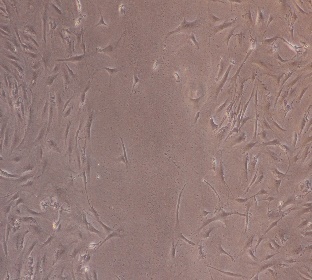 | 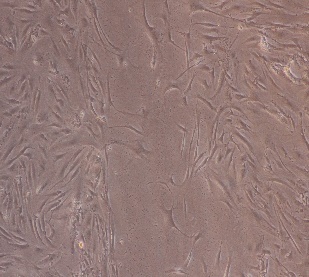 |
|  |  |  |  |  |
| **Day 3** | 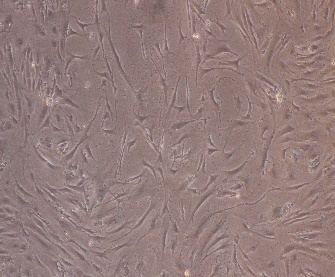 | 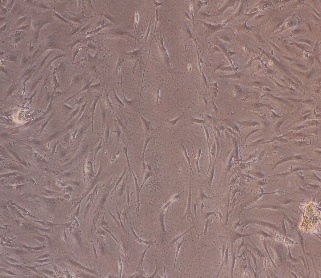 | 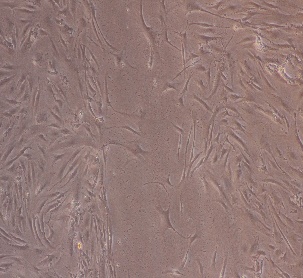 | 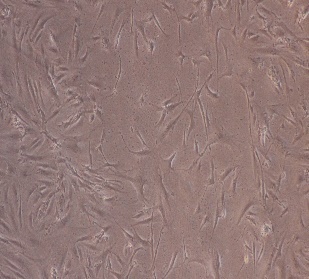 |

**Figure S3** Migration of the gingival fibroblast cells after being incubated with the tooth gel formulations at various time points (mean±s.d., n=3)

The cell migration was not significantly changed in the presence of the tooth gel, indicating that the tooth gel did not significantly enhance wound healing. (M=α-MG tooth gel; L= LME tooth gel; M + L= α-MG and LME tooth gel).
